# Heels: Et Id Omne Genus

**Published:** 1879-07

**Authors:** Ben. H. Pratt


### THE BISTOURY.

Vol. XV. ELMIRA, N. Y., JULY, 1879. No. 2.

### HEELS: ET ID OMNE GENUS.

BEN. H. PRATT.

The heel of a shoe seems a little matter. The chunk of leather that lifts the hinder portion of the foot, is a trifle too insignificant, apparently, to require attention. A variation in height or width ought not, one would at first thought imagine, to call forth so serious a thing as an article in a magazine. Yet is not a little matter, when viewed from the proper standpoint.

The heel of our shoe is not only one of the doubtful improvements upon nature, but it is one of the deformers of our bodies—one of the detractors from a graceful carriage—one of the sources of unhealth—one of the annoyances of our lives—one of those things which go toward making existence a burden—a cause of accidents innumerable—a folly which has less excuse than almost any other within the range of experience.

The human foot is one of the most wonderfully constructed pieces of mechanism to be found in the human body. The anatomist recognizes it as such. He will demonstrate this fact for you if you but give him the opportunity. Such demonstration would occupy too much space to admit of being given here. Besides, one needs the aid of a skeleton and manakin to properly and intelligently accomplish this. An observation of the nice articulation of the numerous and multiform bones of the foot, their dependence upon each other, their relations one to another, their delicate adjustments each with its fellow, and the beautiful adaptation of every little osseous fragment, however queer in shape, to the purpose for which the whole is designed, will speedily convince one of the fact that it is not only unwise to attempt to improve upon it by artificial additions, but that it is a work of supererogation. The muscular structure is equally perfect for accomplishing the purposes designed, and any derangement of such exquisite adaptation of bone and ligament and tendon and muscle is simply a reflection upon the wisdom of the Creator, and a boldness of presumption which only ignorance of the most abject sort can excuse.

To walk with perfect ease—to maintain a graceful carriage without consciousness thereof—to walk naturally and suffer no twinges of pedal pain—one needs but use his feet as they were meant to be used: protected, but not bound, nor stilted, nor cramped, nor pitched forward, nor pinched. The sandal of the ancients is the most sensible thing in the way of foot wear that was ever invented, but inasmuch as the aim of the Bistoury is ever to advocate the practical, and as I have no hope, even the remotest, that this or future generations will ever become so absolutely sensible as to return to this primitive shoe, I must adhere to the possible, and plead for the best the age will be likely to accept.

If we would consult our comfort, not one of us would ever wear another heel upon our shoes. Nature cries out against it. No one can have a natural foot and wear a heel, however low. We were not made to wear heels, otherwise heels would have been put upon the feet. It was never intended that human kind should go plumping along with the hinder part of the foot in the air, and the ankles at any other than a right angle with the feet. We can't balk nature and get any good out of the balk. We can't improve on the make up of a perfect man or woman. We try it and suffer, but we keep on trying just the same. We squeeze the waist and compress the bowels and disturb the abdominal and thoracic organs, and have lung, and heart, and liver, and kidney, and stomach, and bowel, and uterine difficulties, and know that we are shortening our lives every tug we give a corset string or a belt-buckle or other modern body squeezer, but go on hitching ourselves a little tighter each day until we hitch into the grave. We squeeze all our blood into our brain and go to doctors to find out why we have so much headache. We put garters on our legs to keep our stockings tidy, and wind them a little tighter every day until we cut a ridge in our popliteal region that even goes into the bone, and then wonder why we suffer with cold feet, and have cramps, and can't get up any circulation in our extremities; but still we keep right along winding strings and clasping elastics around our legs, defiant of every known law of nature.

Just so we prop up our heels and change the function of walking into a hobble. We go teetering about on our toes, with our feet on an inclined plane, and our toe-bones all rammed into a bunch, and joints sticking out everywhere, to be rubbed into bunions and corns and blisters and all sorts of chiropodical torments. There isn't one man or one woman in a hundred that dares to show his or her bare foot to one of his or her own sex, because he or she has deformed it into such a mean, contemptible, ungainly looking thing that an ape would scorn to own it. Even a shoe heel an inch high will deform a foot, and an inch high heel is an exceedingly low one now-a-days. If it be broad and flat, and the sole is half an inch high, perhaps little harm would come of it. Nature will stand a little meddling with. Then one's ankles don't flop over and crack, and the relations of the foot and leg are kept somewhere near the bounds of normality. Men have of late come down to something akin to sense in this particular, but women? Why, it's perfect torture to see a fashionably heeled woman walk now. They go along clip, clip, clip—heels on a couple of black pegs of dice box pattern—ankles flexed to a perfect strain—careful lest the stilt may not come down square, and the ankle get a wrench—clip, clip, clip—she steps on a pebble and the marks of pain fly athwart her face—toes all in a bunch—corns all twinging—bunions all aching—insteps burning into the leather—why, it's an outrage, the like of which, if inflicted by other than herself, would not be borne an instant.

Then it always seemed as though a lady ought to be more graceful of gait than a man, but just watch her. Shoulders on a see-saw, one up and 'tother down—back all of a wriggle as though something was biting her between the shoulder blades—gait a sort of swing-and-jerk, as though the knees went slowly back to a certain angle and then straightened out suddenly like the blade of a stiff-springed jack-knife—elbows partaking of the same motion, sympathetically—neck poked forward and back, after the manner of some birds whose ballast is in their heads—and all because they are in a sort of accustomed agony. They get used to it, like a boy with a rag on his toe, and his toe sticking up in the air, like a turtle's head. He can smile and forget that his toe knuckle is forty-five degrees askew, and that he is hippity-hoppiting on the northwest corner of his foot, and cutting a deuce of a figure, but he's a boy, and nobody would mind if he was scooting along on his chin, and each finger and toe was pointing to an undiscovered planet in space. But we expect a little something in the way of graceful carriage in a lady, and it is, to say the least, rather mortifying to see her jerking along like an automaton, and not a very good automaton either.

Then one rather looks for quietness of proceeding in ladies. It isn't natural to think of them as clippers, or stampers, or any sort of rattlete-bangers. But they go it with such a clatter on their high heels that one can't tell their steps from that of men. A woman casts half her attractions aside when she becomes pronounced, and loud, and she couldn't do anything that makes her so obtrusive as that peculiar crack which her heels make upon the walk or floor. It is difficult to conceive how a really modest lady can reconcile herself to such an anomaly as heralding her coming with a noisy pair of heels. It looks as though she would like to have people know that she has heels, but fears they won't find it out—as though she wanted to attract attention to her feet, in lieu of other attraction. It's all of a piece with those other devices that flashy women adopt to attract. When women wore plain white hose, and boots without heels, and plain skirts, and bonnets that protected the head, and sleeves to their dresses, instead of to their gloves, and didn't swing their hands, nor wear pockets behind, they were a sort of demi-goddesses. There wasn't any flash about them then, and it seems as though they were a deal more womanly than now. They didn't carry their skirts on their arms, nor pull them into pantaloon-shape, nor suggest any notion that they were not modest.

What can they think, if they think about it at all, as to the impression they create in the minds of well meaning men? Do they expect that all this display goes for nothing? It's no use to mince matters. If we write as they dress, surely they can't find fault. Those carefully modeled heels are certainly for a purpose. They are made to be seen, and good care is taken that they shall be seen. The shoe is the most nicely-selected article of a lady's toilet. She can go frowzy-headed, but her boot must be just so. She goes farther, and dons striped stockings of the most striking hues. These she takes good care to show, also; not fairly and squarely, and as though she meant it, but nippingly, mincingly, now a little bit, now a little more, then hides them altogether; then flashes half a yard of striped hose at you, as much as to say, "Now you see it, and now you don't." Then is it not too baldly apparent that ladies now trim their dresses in anything but a modest way—in fact, the most immodest way consistent with decency? Why are the loopings and puffings, and plaitings and other varieties of that multitude of mysteries known as trimmings, all concentrated upon that portion of the dress which the wearer blushes to speak of, if she isn't too modest to speak of it at all? If it be not to attract attention to that particular part of the garment, the fact that these are there is sufficient to warrant the beholder in supposing that it is; and no one can draw any other inference therefrom.

The argument that such is the mode, and that one must wear her garments so or become peculiar aud marked in her avoidance of the fashion set her by the authorities in these matters, is an exceedingly lame one. If modesty does not enter into the question; if they who set the fashion after which one must model her garments, disregard the proprieties so far as to lay the motive open to suspicion; if fashion dictates that to appear in a raiment that suggests a wish to reveal, beyond what is meet, that portion of the person which common decency claims should be concealed from view; then there is but one of two things to be done—accept the situation and expose one's self to the suspicion of an immodest motive, or defy the edict and retain one's womanhood at all hazards.

It is a fearful thing to believe that the women whom in every other particular we admire; who have, in everything save dress, the most excellent judgment; for whose opinions on all subjects within their sphere we have the very highest regard, to whom we cheerfully commit all questions of propriety, confident that their decisions are a sufficient guide; that these same women will actually conspire to arouse, in the minds of those over whom they know they have a weighty moral influence, a suspicion that their teachings, their precepts and their home example are but a sham, because their actions, as exhibited in their dress, belie their character and compromise their principles.

A lady with a dress shamefully low in the neck—immodestly lacking in the sleeves—suggestively lifted to an improper height—shamelessly padded to a highly improbable contour—so outrageously puffed and conspicuously looped over a portion of her anatomy to which no delicate woman would for a moment otherwise call attention—so wantonly drawn back over her thighs as to reveal the figure beyond peradventure; a lady who dresses her feet for show—who commands attention to her limbs by donning stockings of loudest hue—who perches herself upon heels of monstrous height and dangerous delicacy—who apes the courtesan in wearing her hair in the form of the idiotic "bang;" in a word, believes she can throw out all the signals of immodesty and yet retain the genuine respect of her honest friends; all such make a sad mistake, and it is among the most wonderful of phenomena that they cannot,—for they claim that they cannot—see themselves in this light.

Running into analogies has taken us away from our subject, but it's all of a piece, this showy and pronounced way of dressing, and whether we discourse of heels or other parts of the body, the tendency is all the same. There can be but one conclusion, and this is that for the sake of doing as others do, and dressing as others dress, and displaying as full a complement of attractions as anyone else, women will sacrifice that which is of priceless worth to them, the good opinion of their most ardent friends. I care not how highly a woman may be esteemed, he who witnesses the compromises she makes with modesty by adopting the style of the immodest, cannot maintain for her such an exalted opinion as he wishes he could. She steps down from her pedestal and becomes of the common, and no defense she can make will ever lift her to her former place in his esteem.

What, then, is the demand now loudly made for the reclamation of women from a suspicion of immodesty—from a defiance of the laws of health—from premature decline—from a tendency to become corrupt—from being a source of chagrin to all who entertain for them a genuine respect? It is that there arise some woman who has strength enough, and purpose enough, and force enough, and influence enough, and enough of fanaticism to carry her to, and not beyond, the boundary line of feminine gentility and womanly self-respect—that there arise such a woman, who will dare defy the arts and resist the wiles of the ultra-fashionists, and show that it is not womanly to do as women do, but that she is that true and noble one among women, who consults, first her conscience, then her promptings as to what is decent and becoming in her, and, finally, the desire rather to charm and attract to herself that class of friends who are drawn into her society and her confidence rather by her purity of heart and chastity of intellect than by those more pronounced but transparent brilliants of dress and carriage which may be so readily put on and off at will, and in which the vilest of her sex can be her equal upon an hour's notice.

There is just one other point I wish to make right here, and that is the blame which properly attaches to men, and especially to young men, for encouraging this propensity in young women to dress after a fast style. It is too often the case that young men—and some silly older ones—encourage women in their loud dressing. It is considered so desirable that ladies with whom they associate should appear as stunningly dressed as possible, and be gotten up nobbily, as they express it. To be seen with a "plain" girl is felt to be quite derogatory to the position of the average young man. The gay are run after and taken everywhere to be shown off, and if she adds to her feathery, flouncy, flashy, flaunting dress, a corresponding frothiness of talk and a not over particular modesty of manner, she is voted gay—in the crowd at the bar or billiard-room, after an evening of rather *blase* sociability. It is a sad thing that men should so aid in making popinjays of women, and it is a temptation that women find hard to resist, this dressing and overdressing and trick-dressing to be admired. But these men should remember that they are educating wives, either their own or another's, and that somebody else is perhaps educating their's in all this fearful vanity. On the whole, I am not sure but that the major part of the blame for what we daily see of this extravagant dressing, and conspicuous dressing, and dressing suggestive of immodesty, attaches to the very sex that in its sober moments so bitterly inveighs against what we have been attempting to show as a fearful laxness in the dress of the women of to-day.

It is worth thinking over, and pondering upon, and making good resolutions about. If men cease to encourage flash, the desire to be flashy will assuredly decline. If the exceptionally proper receive the same attention as do those exceptionally inclined in the opposite direction, the temptation to be unwomanly will have ceased. It is worth trying for, and unless the underlying idea of this article is diametrically wrong, then we may witness a most decided, and surely an agreeable change in the notion of what constitutes charming women.

